# Ultrasonographic diagnosis of splenic torsion: a case report

**DOI:** 10.3389/fsurg.2025.1586986

**Published:** 2025-06-19

**Authors:** Zhiyan Di, Shasha Zhao

**Affiliations:** Department of Function, Baoding Hospital, Beijing Children's Hospital Affiliated to Capital Medical University, Baoding, Hebei, China

**Keywords:** splenic torsion, pediatric ultrasound, whirlpool sign, wandering spleen, emergency

## Abstract

We report an exceptional case of 900° splenic-torsion with accessory spleen in a 17-month-old girl presenting with nonspecific vomiting and fever. Initial CT suggested an abdominal mass, ultrasonography definitively diagnosed splenic torsion through characteristic findings: absent parenchymal flow, twisted hilar vessels (“whirlpool sign”), and preserved accessory spleen perfusion. This case highlights ultrasound's superior diagnostic capability over CT for pediatric splenic torsion, particularly in demonstrating dynamic vascular compromise. The report emphasizes key sonographic criteria and the critical 6-hour window for salvageable splenic ischemia.

## Case presentation

**Figure 1:**
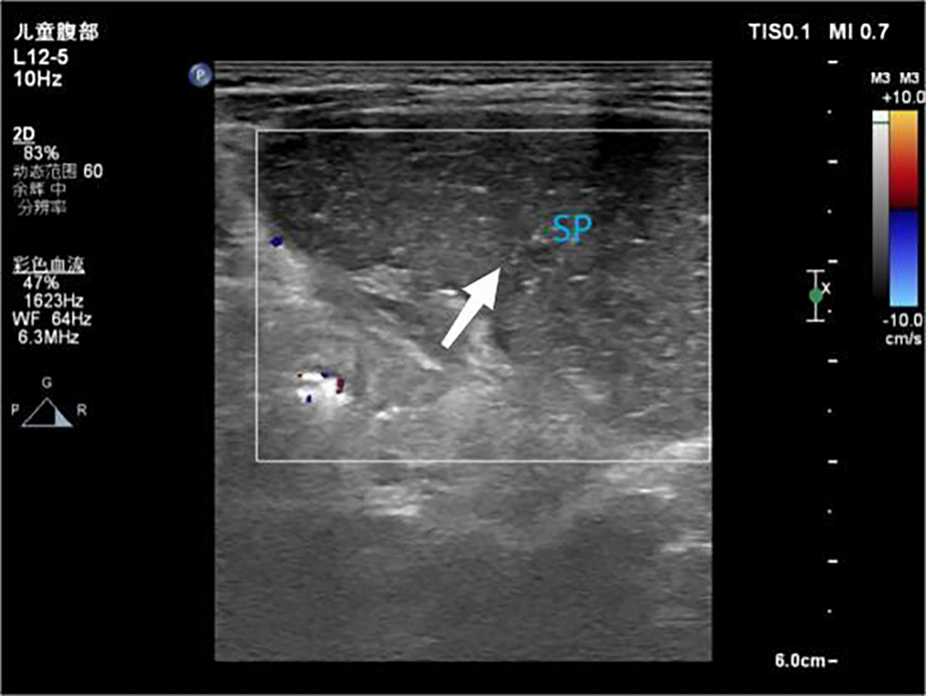
Ultrasound image showing no blood flow signal within the spleen.

**Figure 2:**
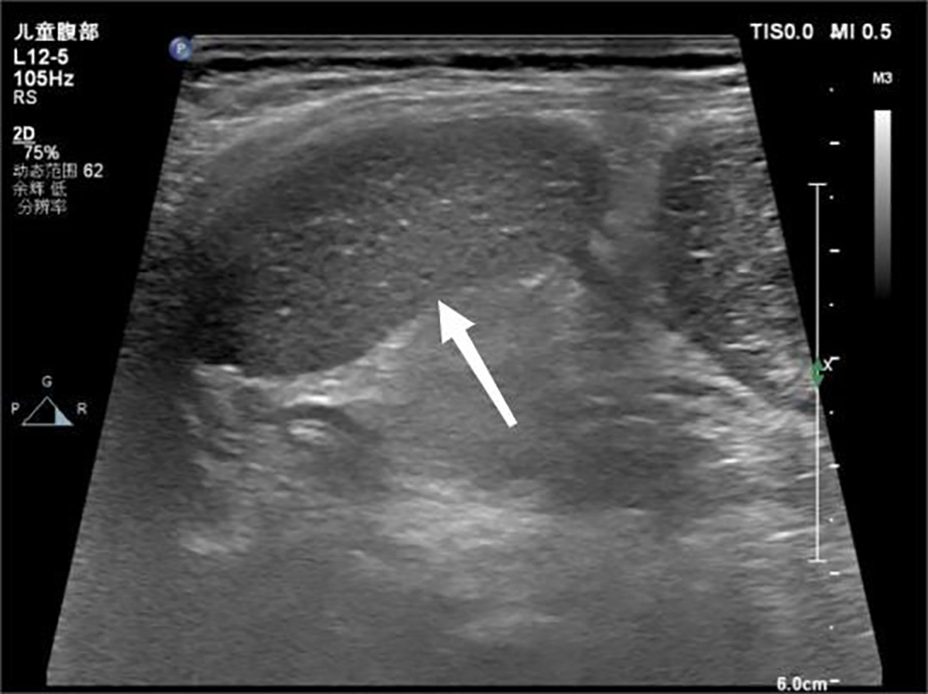
Ultrasound image showing the accessory spleen in the splenic region.

**Figure 3:**
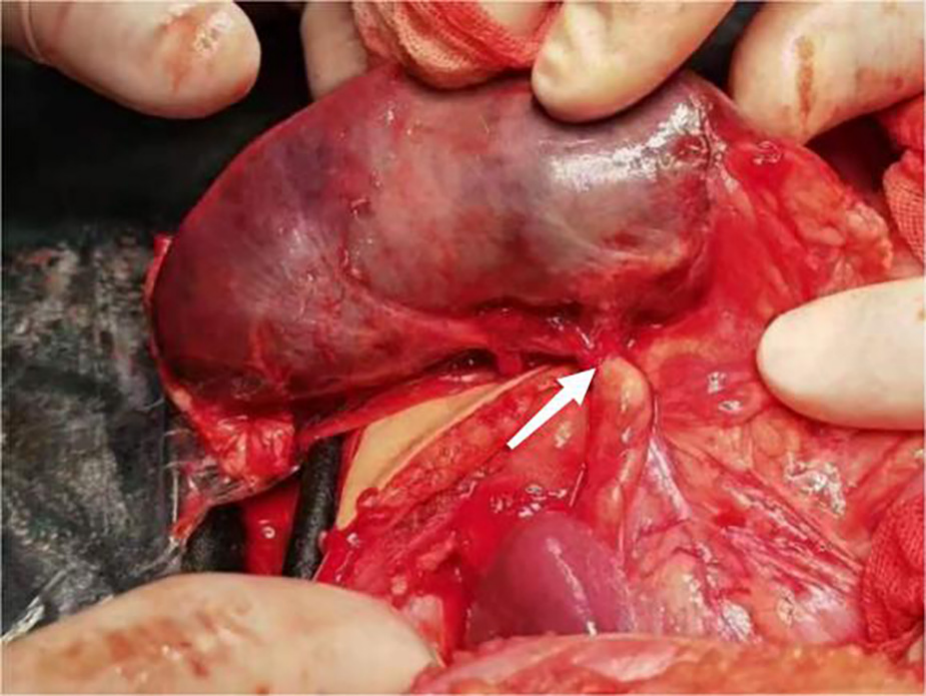
Intraoperative image of the torsed spleen.

A 17-month-old female patient was admitted to Baoding Hospital, Beijing Children's Hospital Affiliated to Capital Medical University after an abdominal mass was identified on computed tomography (CT) imaging. Before admission, the patient had a history of vomiting and fever. Upon admission, the patient's general condition was stable, but she was uncooperative during the physical examination. There was no tenderness upon palpation of the abdomen. Ultrasound findings revealed a mass in the left upper quadrant of the abdomen, measuring 8.7 × 2.3 × 4.1 cm, with echogenicity similar to that of the spleen but with a coarse texture. No blood flow signals were detected within the mass (Figure 1). A splenic hilum-like structure was observed, with a feeling of torsion at the pedicle. A secondary splenic echo was noted at the splenic level (Figure 2), measuring 4.6 × 2.3 cm, with normal blood supply. The ultrasound diagnosis was splenic torsion with necrosis and an accessory spleen. Intraoperative findings confirmed the diagnosis: the spleen, measuring 9.0 × 3.0 × 4.5 cm, an accessory spleen, measuring 5.0 × 2.0 cm, was located above the main spleen (in the splenic region). The spleen twisted 900° counterclockwise at the splenic hilum, as confirmed intraoperatively (Figure 3). Postoperative pathology confirmed hemorrhagic necrosis of the splenic tissue due to torsion. Follow-up ultrasound showed good blood supply to the accessory spleen.

## Discussion

Spleen torsion is a rare cause of organ infarction accompanied by non-specific acute abdominal symptoms, especially in children, accounting for less than 0.2% of pediatric acute abdominal cases. Our literature review found that less than 50 cases involved the accessory spleen, making this case particularly noteworthy (1, 2). Due to the lack of typical clinical manifestations, it often manifests as abdominal pain, vomiting, and bloating, which overlap with more common pediatric gastrointestinal diseases, leading to significant diagnostic challenges and frequent misdiagnosis (3). The main reasons include splenic wandering, excessive splenic pedicle, or splenomegaly. When there is sudden change in abdominal pressure, posture, or abnormal intestinal peristalsis, it is easy to trigger. Our case exhibits three rare features: extreme 900° rotation (usually 180–360°), simultaneous presence of active accessory spleens, and delayed diagnosis due to non-specific CT findings. Wandering the spleen is a susceptible factor for torsion, caused by congenital laxity or absence of splenic ligaments, leading to abnormal activity (4). During chronic splenic torsion, spleen congestion and enlargement may occur, and children may not have obvious clinical symptoms, only mild abdominal pain, vomiting, nausea, and other symptoms. The abdominal mass found in our case has raised important diagnostic considerations for its potential etiology. Although the surgical results confirmed secondary splenic necrosis after torsion, differential diagnosis must also include free spleen (ectopic spleen) as a potential susceptibility factor. Anatomical factors such as congenital deficiency, laxity, or underdeveloped splenic ligaments can easily lead to splenic torsion, highlighting that free spleens may present as abdominal masses before torsion occurs. The importance of early imaging suspicion, as delayed diagnosis (>24 h) can lead to irreversible infarction or spontaneous rupture, requiring splenectomy. Initially, the venous return to the spleen was damaged, leading to congestion and enlargement of the spleen. When arterial blood flow is interrupted, ischemic necrosis and inflammatory exudation occur, leading to local peritonitis (3). The characteristics of splenic torsion pose a challenge to diagnosis. Preoperative diagnosis of splenic torsion is often difficult to achieve, as neither ultrasound nor CT scans can accurately distinguish between splenic infarction, abscess, or tumor (5), and there is no definitive report on the sensitivity and specificity of ultrasound in diagnosing splenic torsion. Ultrasound is a reliable diagnostic tool for splenic torsion and should be considered the first-line imaging modality (1, 6). When splenomegaly or changes in splenic position are noted, attention should be paid to the vascular condition of the splenic pedicle and splenic parenchyma. If a mass of blood vessels at the splenic hilum or a “whirlpool sign” is observed, along with reduced or absent blood flow in the splenic parenchyma (7), or signs of infarction, splenic torsion should be considered. In this study, splenic torsion was diagnosed by ultrasound. When ultrasound cannot provide a clear diagnosis, it is recommended to further perform enhanced CT to assist in diagnosis. However, there is no clinical comparison between ultrasound or CT in diagnosing splenic torsion. Generally, ultrasound examination is preferred because it is fast, expensive, and radiation free. However, the 17 month old girl in this report only underwent CT plain scan and did not undergo enhanced CT due to parental opposition, and no splenic vascular distortion was observed, which may be the main reason for the unclear diagnosis of CT.

The golden period for diagnosing splenic torsion is within 6–12 h after onset, which can increase the possibility of preserving the spleen. Delayed diagnosis (>24 h) may lead to irreversible splenic infarction, spontaneous rupture, or infection (such as splenic abscess), requiring splenectomy. However, delays may occur due to atypical symptoms of splenic torsion, poor coordination with physical examinations in children, or limitations in examinations (such as avoiding CT scans). Diagnostic delays are typically caused by non-specific manifestations of the disease, challenges in examining pediatric patients, and reluctance to use advanced imaging techniques in young children. These factors collectively emphasize the importance of maintaining a high suspicion index for splenic torsion when evaluating pediatric patients with unknown abdominal symptoms, especially when a physical examination shows palpable abdominal masses.

## Data Availability

The raw data supporting the conclusions of this article will be made available by the authors, without undue reservation.
