# Supplementary figures and images for: Ultrasonographic diagnosis of splenic torsion: a case report
Source: Front Surg. 2025 Jun 19;12:1586986. doi: 10.3389/fsurg.2025.1586986 (PMC12222168; doi:10.3389/fsurg.2025.1586986)

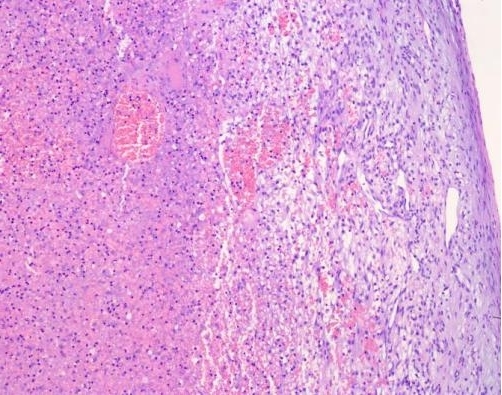

Supplement: Supplementary file 1 [file Image1.jpeg]
